# Exploring the role of aggrephagy-related signatures in immune microenvironment, prognosis, and therapeutic strategies of breast cancer

**DOI:** 10.1097/MD.0000000000039999

**Published:** 2024-10-18

**Authors:** Chenbo Ye

**Affiliations:** a Department of Breast and Thyroid Surgery, Shaoxing Shangyu Hospital of Traditional Chinese Medicine, Shangyu, Zhejiang, China.

**Keywords:** aggrephagy, Breast cancer, drug sensitivity, immune infiltration, prognosis

## Abstract

Breast cancer (BC) is 1 of the most common malignant tumors among women globally. This study aimed to develop a prognostic signature based on aggrephagy-related genes (ARGs). Transcriptomic and clinical data for BC patients were downloaded from the cancer genome atlas and GEO databases. Differential expression analysis, univariate Cox proportional hazards regression and least absolute shrinkage and selection operator Cox regression were employed to construct a prognostic signature. Consensus clustering, evaluation of immune infiltration and drug sensitivity, and gene set enrichment analysis, and development of nomogram were performed. The expression of ARGs was validated using data from the Cancer Cell Line Encyclopedia and clinical samples. Eleven ARGs were abnormally expressed in BC, with 5 showing significant correlations with BC prognosis. Consensus clustering identified 2 molecular subtypes with distinct prognoses. A prognostic signature including 5 ARGs (*VIM*, *TUBB1*, *TUBA3E*, *TUBA3D*, *TUBA1C*) was developed, which showed high performance in predicting BC prognosis. The low-risk group showed enrichment in extracellular matrix organization and cell migration processes while chromosome separation was suppressed. Additionally, patients in this group also show activation in several signaling pathways including MAPK, PI3K-AKT, and cAMP pathways, whereas cell cycle and neutrophil extracellular trap formation were significantly inhibited. The signature was also associated with immune infiltration and drug sensitivity. A nomogram incorporating the risk signature, clinical stage and chemotherapy was constructed, demonstrating excellent performance in predicting prognosis. The expression of signature-related genes were validated in patients with BC. This study successfully constructed molecular subtypes and a prognostic signature based on ARGs in BC, and developed a nomogram.

## 1. Introduction

Breast cancer (BC) constitutes a prevalent malignancy among women globally, accounting for an annual incidence rate of 11.67% among all newly diagnosed cancer cases, wherein it further contributes to 6.69% of total cancer fatalities annually.^[1]^ Its complex etiology is characterized by a multifactorial interplay between genetic predispositions, hormonal influences, lifestyle factors, and environmental exposures.^[2]^ The clinical course of BC can vary significantly, necessitating the development of more precise risk stratification models to guide personalized treatment strategies and improve patient outcomes.^[3]^ Despite advances in early detection methods and therapeutic interventions, there remains a critical need for novel biomarkers and pathophysiological insights that could better predict disease progression, response to therapy, and overall survival.^[4]^

Aggrephagy, an autophagic process dedicated to the clearance of protein aggregates, has emerged as a pivotal cellular homeostatic mechanism.^[5]^ Protein aggregates are regulated by phase separation during their formation, transitioning between various states such as liquid and solid, which influences the nature of the aggregates and their clearance methods.^[6]^ The formation of protein aggregates can generate new protein toxicities, which, in addition to losing their normal functions, can interact with other properly folded proteins, causing intracellular protein function disorder.^[6]^ Dysregulation of aggrephagy is increasingly implicated in various diseases, including neurodegenerative disorders and cancers.^[7,8]^ Recently, risk profiles built upon aggrephagy-related genes (ARG) have been constructed in multiple cancer types and have demonstrated correlations with tumor prognosis, the tumor immune microenvironment, and drug resistance.^[9–11]^ However, their value in predicting BC prognosis remains underexplored.

**Figure 1.**
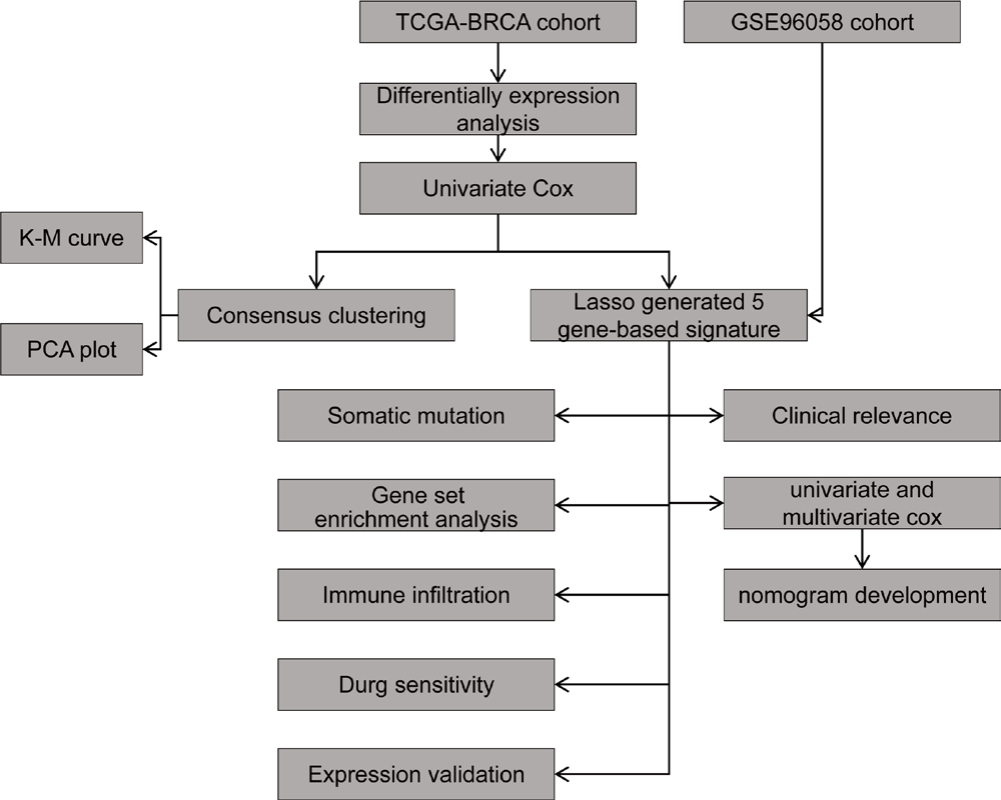
The workflow diagram of this study.

This study aims to explore the potential role of ARGs in determining BC risk and prognosis. By integrating multi-omics data and utilizing cutting-edge bioinformatic approaches, we aim to identify a distinct signature of ARGs associated with BC risk and aggressiveness. Our research objectives include the construction of a novel risk profile based on the expression patterns of these ARGs and the assessment of its predictive value in terms of patient prognosis and response to treatment. Ultimately, this work seeks to elucidate the functional significance of aggrephagy dysregulation in BC biology and to provide a foundation for the development of targeted interventions that exploit this pathway’s therapeutic potential. The workflow diagram was illustrated in Figure 1.

## 2. Materials and methods

### 2.1. Data acquisition and processing

We obtained RNA sequencing data and comprehensive clinical-pathological information for the breast invasive carcinoma (BRCA) project from the cancer genome atlas (TCGA) database. Standardized RNA-seq expression quantification data and corresponding clinical annotation files were downloaded through TCGA’s genomic data commons data portal (https://portal.gdc.cancer.gov/). Cases missing critical clinical information were excluded. Furthermore, to ensure the validity and reliability of survival analysis, cases with a prognosis time <30 days were removed from the analysis set to minimize bias introduced by short-term follow-ups. To validate our analysis results or expand the sample scope, we also retrieved the publicly archived GSE96058 dataset from the Gene Expression Omnibus (https://www.ncbi.nlm.nih.gov/geo/) database, which contains transcriptome data and associated clinical-pathological information for 3406 BC patients. Forty-four ARGs were obtained from previously published literature (Table S1, Supplemental Digital Content, http://links.lww.com/MD/N722).

### 2.2. Consensus clustering analysis

Differential expression analysis was performed using the edgeR package to identify ARGs with *P* < .05 and |log2 (fold change)| >1. Somatic mutations were analyzed using the maftools package. Univariate cox regression analysis was employed to assess the association between ARGs and BC prognosis, selecting ARGs with *P* < .05 for consensus clustering analysis. The ConsensusClusterPlus package was used to conduct the clustering analysis, employing the partitioning around medoids method and the “Pearson” distance function, followed by K–M survival curve analysis to evaluate prognosis differences among subtypes.

### 2.3. Construction of prognostic model

A least absolute shrinkage and selection operator (LASSO) cox regression analysis was conducted using the glmnet package to build a risk signature. The calculation formula is as follows: risk score = ∑(βi * expi), where βi represents the coefficient of gene i, and expi denotes the expression level of gene i. Based on the median value of risk score, high-risk and low-risk groups were divided and subjected to K–M survival analysis and receiver operating characteristic (ROC) curve analysis.

### 2.4. Tumor microenvironment analysis

Tumor microenvironment analysis was conducted using the IOBR package, which encompasses various methods for evaluating immune infiltration in tumors, including CIBERSORT, ESTIMATE, and IPS.

### 2.5. Drug sensitivity analysis

Drug sensitivity analysis was performed using the pRRophetic package to analyze the sensitivity of TCGA-BRCA cohort patients to 45 drugs, including Axitinib, Bexarotene, Bicalutamide, Bleomycin, and Bortezomib, among others. Sensitivity assessment utilized the pRRopheticPredict() function with tissueType set to “all,” and batch correction was applied using the ComBat method.

### 2.6. Gene set enrichment analysis

Differential expression analysis was carried out using the edgeR package, followed by gene set enrichment analysis (GSEA) utilizing the clusterProfiler package, covering both gene ontology and Kyoto encyclopedia of genes and genomes datasets.

### 2.7. Somatic mutation analysis

Somatic mutation analysis was conducted using the maftools package. The top 10 most frequently mutated genes in the high- and low groups were visualized using the oncoplot function. A comparison of tumor mutational burden (TMB) differences between the 2 groups was performed, and the correlation between the risk score and TMB was analyzed. Patients were further divided into high TMB (HTMB) and low TMB (LTMB) groups based on the median TMB value, and Kaplan–Meier survival analysis was performed for these groups.

### 2.8. Construction and evaluation of nomogram

Univariate and multivariate cox regression analyses were employed to assess the prognostic value of risk score and clinical-pathological characteristics. Independent prognostic factors with *P* < .05 were selected, and a nomogram was constructed using the rms package to predict the 1-, 2-, and 3-year overall survival for BC patients. The performance of the nomogram was evaluated through ROC curve analysis, calibration curve analysis, and decision curve analysis using the rmda package.

### 2.9. Analysis of the expression of aggrephagy-related genes in breast cancer

In order to elucidate the expression patterns of ARGs in BC, we retrieved their expression levels across prostate cancer cell lines and normal cell lines from the cancer cell line encyclopedia (CCLE; accessible at https://depmap.org/portal/). Subsequently, we conducted a comparative analysis of ARGs expression between normal tissue samples and cancerous tissue samples from The TCGA dataset to further delineate their expression profiles within the context of BC.

### 2.10. Sample collection

Breast cancer specimens and corresponding adjacent normal tissues were collected from 5 patients. Immediately after procurement, the samples were snap-frozen in liquid nitrogen and stored at −80°C for subsequent analysis. Comprehensive informed consent was obtained from all participants before sample collection. Approval for this study was granted by the Ethics Committee of the Department of Breast and Thyroid Surgery at the Shaoxing Shangyu Hospital of Traditional Chinese Medicine (approval number: 2024-16).

### 2.11. Quantitative real-time polymerase chain reaction

RNA extraction was performed using TRIzol reagent (Thermo Scientific, USA) according to the manufacturer’s instructions. Two micrograms of RNA were utilized for cDNA synthesis with the RevertAid First Strand cDNA Synthesis Kit (Thermo Scientific, USA). Quantitative real-time PCR (qRT-PCR) was conducted using SYBR Green Mix (Vazyme, Nanjing, China) to assess the mRNA levels of ARGs. The quantitative data were normalized to *B2M*. The primer sequences used in the experiments are provided in Table 1.

**Table 1:** Primer sequences for qRT-PCR assay.

Genes	Forward (5′-3′)	Reverse (5′-3′)
*VIM*	TGAAGGAAGAGATGGCTCGT	TCCAGCAGCTTCCTGTAGGT
*TUBB1*	GGGACGATGGACAGCATTCGAT	ACCTCTAGGACATTCTCGATCAGC
*TUBA1C*	CCGGGCAGTGTTTGTAGACT	TTGCCTGTGATGAGTTGCTC
*TUBA3D*	AGGCATGGAAGAGGGAGAGT	TGCAAACAACTTGGAAGCAG
*TUBA3E*	GTGGATTCCGTGGAAGCTGA	GAAAGCAGCCATCCTAGGGG
*B2M*	GAGGCTATCCAGCGTACTCCA	CGGCAGGCATACTCATCTTTT

### 2.12. Statistical analysis

All data analyses and visualizations were conducted using R software (version 4.4.1). Between-group differences were assessed using the Wilcoxon test, with *P* < .05 considered statistically significant. Kaplan–Meier survival curves and Log-rank tests were executed using packages such as survival and survminer for survival analysis.

## 3. Results

### 3.1. Aggrephagy-related gene-derived molecular subtypes in breast cancer

**Figure 2.**
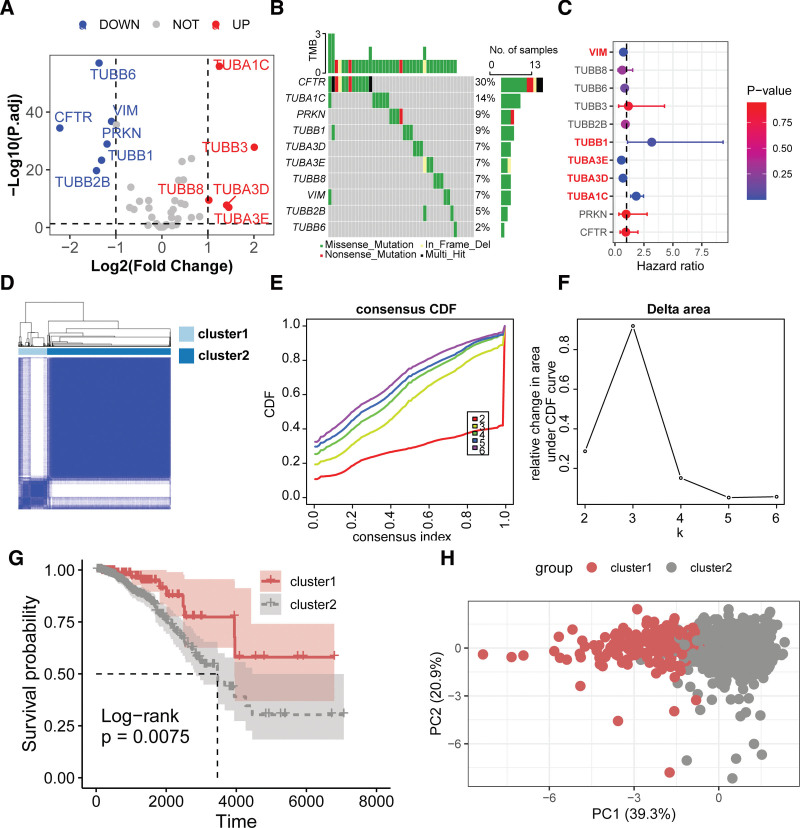
Expression, mutation, prognostic significance, and stratification ability of aggrephagy-related genes (ARGs) in the cancer genome atlas (TCGA)-BRCA cohort. (A) Volcano plot illustrating the differential expression of ARGs in breast cancer (BC). (B) Top 10 genes with the highest frequency of mutations among ARGs. (C) Hazard ratios of differentially expressed ARGs associated with breast cancer prognosis. (D–F) Consensus clustering analysis based on prognosis-related ARGs. (G) Kaplan–Meier survival curves comparing the prognosis difference between molecular subtypes derived from ARGs. (H) Principal component analysis based on prognosis-related ARGs.

Differential expression analysis revealed that 11 ARGs were aberrantly expressed in BC, with 5 genes (*TUBA1C*, *TUBB3*, *TUBB8*, *TUBA3D*, *TUBA3E*) overexpressed and 6 (*TUBB6*, *VIM*, *CFTR*, *PRKN*, *TUBB1*, *TUBB2B*) underexpressed (Fig. 2A). *CFTR* displayed the most frequent somatic mutations, followed by *TUBA1C* and *PRKN* (Fig. 2B). Univariate Cox analysis indicated that among the 11 differentially expressed ARGs, 5 were significantly associated with prognosis in BC (Fig. 2C), including *VIM*, *TUBB1*, *TUBA3E*, *TUBA3D*, and *TUBA1C*. Consensus clustering based on these prognosis-related ARGs resulted in 2 distinct molecular subtypes of BC (cluster1 and cluster2) (Fig. 2D–F), which showed significantly different outcomes (Fig. 2G, *P* = .0075). Principal component analysis demonstrated a clear separation between cluster1 and cluster2 (Fig. 2H).

### 3.2. Risk score based on aggrephagy-related genes

**Figure 3.**
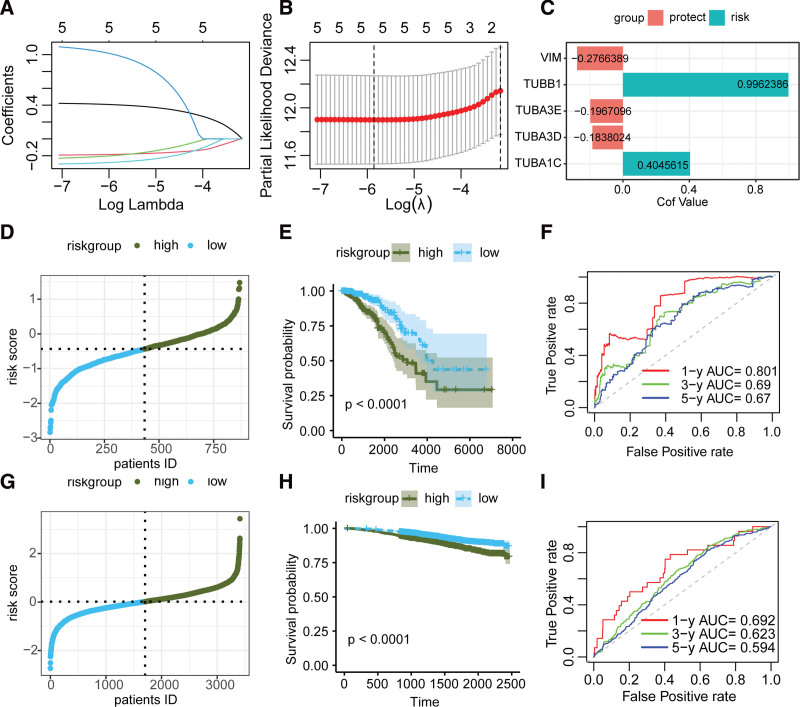
Construction and Evaluation of the ARG-derived Risk Signature. (A and B) LASSO cox regression analysis used to construct the ARG-based risk model. (C) Coefficients of the 5 genes in the model. (D–F) Survival analysis and evaluation of predictive performance using ROC curves in the TCGA-BRCA cohort, where patients are stratified into high- and low-risk groups according to the risk signature. (G–I) Survival analysis and evaluation of predictive performance using ROC curves in the GSE96058 cohort, where patients are stratified into high- and low-risk groups according to the risk signature.

Subsequently, we constructed a risk model using LASSO Cox regression analysis incorporating the 5 prognosis-related ARGs, indicating no redundant genes (Fig. 3A and B). Figure 3C shows the coefficients for each gene within the risk score, with the calculation formula for risk score being: risk score = −0.2766389 * *VIM *+ 0.9962386 * *TUBB1*−0.1967096 * *TUBA3E*−0.1838024 * *TUBA3D *+ 0.4045615 * *TUBA1C*. Based on the median value, patients were stratified into high-risk (n = 435) and low-risk (n = 435) groups in the TCGA-BRCA cohort (Fig. 3D). The mean risk score values were −0.0098 and −0.9299 for the high-risk and low-risk groups, respectively. The high-risk group exhibited significantly worse prognosis compared to the low-risk group (Fig. 3E). In the GSE96058 cohort, patients were similarly divided into low-risk (n = 1705) and high-risk (n = 1704) groups. The mean risk score values were 0.4406 and −0.4233 for the high-risk and low-risk groups, respectively. The low-risk group showed a more favorable prognosis compared to the high-risk group (Fig. 3G and H). ROC curve analysis revealed that the area under the curve (AUC) values for predicting 1-, 3-, and 5-year overall survival by risks core were 0.801, 0.69, and 0.67, respectively, in the TCGA-BRCA cohort (Fig. 3F), and 0.692, 0.623, and 0.594, respectively, in the GSE96058 cohort (Fig. 3I).

### 3.3. Clinical relevance of the aggrephagy-related gene-based risk signature

**Figure 4.**
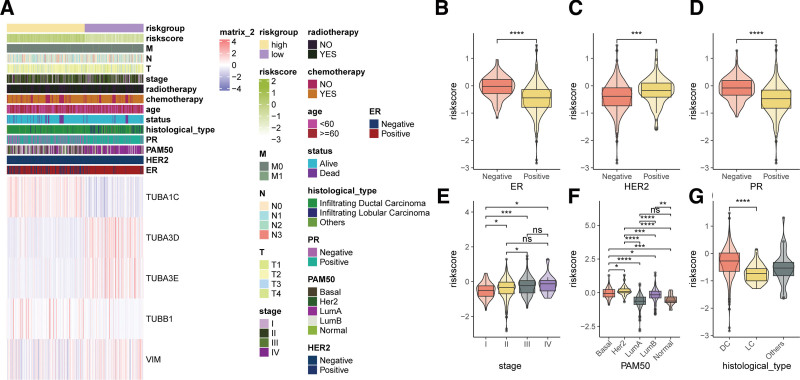
Relationship between the ARG-derived risk signature and clinical-pathological characteristics. (A) Heatmap showing the expression of genes related to the ARG-based risk signature. Comparison of risk scores between different (B) ER status, (C) HER2 status, (D) PR status, (E) stages, (F) PAM50 subgroups, and (G) histological types. ns, not significant, *P < .05, **P < .01, ***P < .001, ****P < .0001. ER, Estrogen Receptor, HER2, human epidermal growth factor receptor 2, PR, progesterone receptor, PAM50, predictive analysis of microarrays 50 genes, DC, ductal carcinoma, LC, lobular carcinoma.

To elucidate the relationship between the ARG-derived risk signature and BC clinical-pathological features, we generated an expression heatmap for risk signature-related genes (Fig. 4A). In this heatmap, *TUBA3D*, *TUBA3E*, and *VIM* were found to be downregulated in the high-risk group, while other genes were upregulated. It was observed that patients who were positive for ER or PR had lower risk scores compared to those who were negative (Fig. 4B and D). In contrast, patients who were positive for HER2 had higher risk scores compared to those who were negative (Fig. 4C). Furthermore, the risks core was higher in advanced stage patients relative to early stage patients (Fig. 4E). In the PAM50 subtypes, the risk score for the HER2 subtype is notably higher compared to other subtypes, while the risk score for the LumA and Normal subtypes is the lowest (Fig. 4F). Additionally, patients with ductal carcinoma exhibit a higher risk score compared to those with lobular carcinoma (Fig. 4G). However, no significant associations were observed between the risks core and features such as TNM staging, radiotherapy, chemotherapy, or age.

### 3.4. Somatic mutations associated with risk score

**Figure 5.**
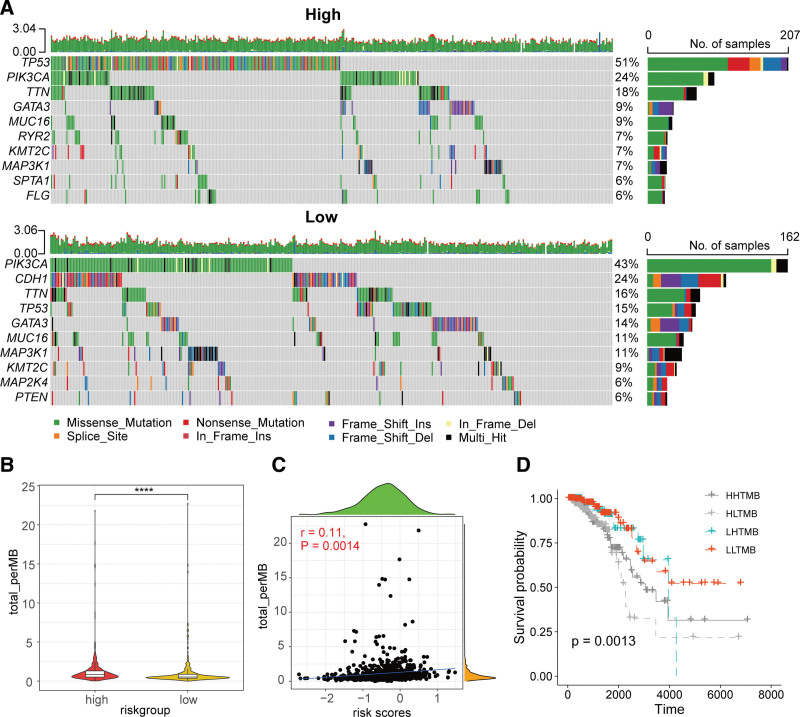
Relationship between the ARGs-derived risk signature and somatic mutations. (A) Oncoplots of the top 10 most frequently mutated genes in the high- and low-risk score groups. (B) Comparison of tumor mutational burden (TMB) between the high- and low-risk score groups. (C) Correlation analysis between TMB and risk score. (D) Kaplan–Meier survival analysis of patients stratified by different TMB and risk score combinations. **** *P* < .0001.

Further analysis was conducted on the somatic mutation profiles in the high- and low groups. In the high group, the top 10 most frequently mutated genes were *TP53*, *PIK3CA*, *TTN*, *GATA3*, *MUC16*, *RYR2*, *KMT2C*, *MAP3K1*, *SPTA1*, and *FLG*. In the low group, the top 10 most frequently mutated genes were *PIK3CA*, *CDH1*, *TTN*, *TP53*, *GATA3*, *MUC16*, *MAP3K1*, *KMT2C*, *MAP2K4*, and *PTEN* (Fig. 5A). Compared to the high group, the TMB values were significantly lower in the low group (Fig. 5B). Correlation analysis revealed a weak positive correlation (*R* = 0.11) between risk score and TMB, which was statistically significant (*P* = .0014, Figure 5C). Kaplan–Meier survival analysis showed no significant difference in prognosis between HTMB and LTMB. Among different TMB subgroups, the low group had better survival outcomes than the high group (Figure 5D, Figure S1). http://links.lww.com/MD/N721

### 3.5. Gene expression regulation related to the aggrephagy-related gene-based risk feature

**Figure 6.**
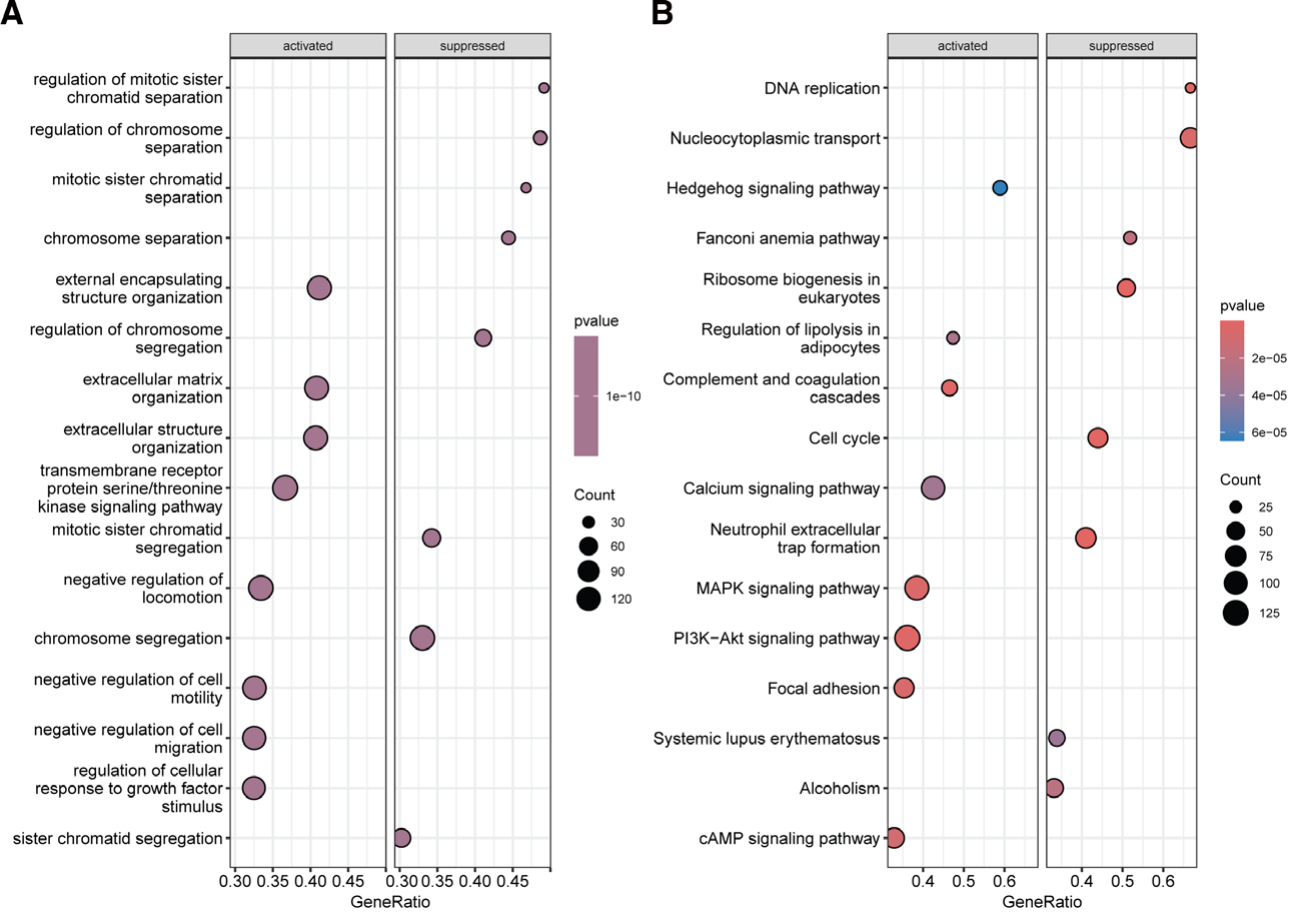
Gene set enrichment analysis between high- and low-risk groups. (A) Gene ontology (GO) term enrichment analysis comparing high- and low-risk groups. (B) Kyoto encyclopedia of genes and genomes pathway enrichment analysis comparing high- and low-risk groups.

To unravel the underlying mechanisms linking the ARG-derived risk feature to BC prognosis, we performed GSEA. The results showed that in the low-risk group compared to the high-risk group, processes such as extracellular matrix organization and cell motility were significantly activated, while the regulation of chromosome segregation and separation was significantly inhibited (Fig. 6A). Moreover, in the low-risk group, several pathways were significantly activated, including the hedgehog signaling pathway, complement and coagulation cascades, calcium signaling pathway, MAPK signaling pathway, PI3K-AKT signaling pathway, and cAMP signaling pathway (Fig. 6B). Conversely, pathways like nucleocytoplasmic transport, cell cycle progression, neutrophil extracellular trap formation, and DNA replication were significantly suppressed in the low-risk group compared to the high-risk group.

### 3.6. Association of the aggrephagy-related gene-based risk feature with tumor microenvironment and drug sensitivity

**Figure 7.**
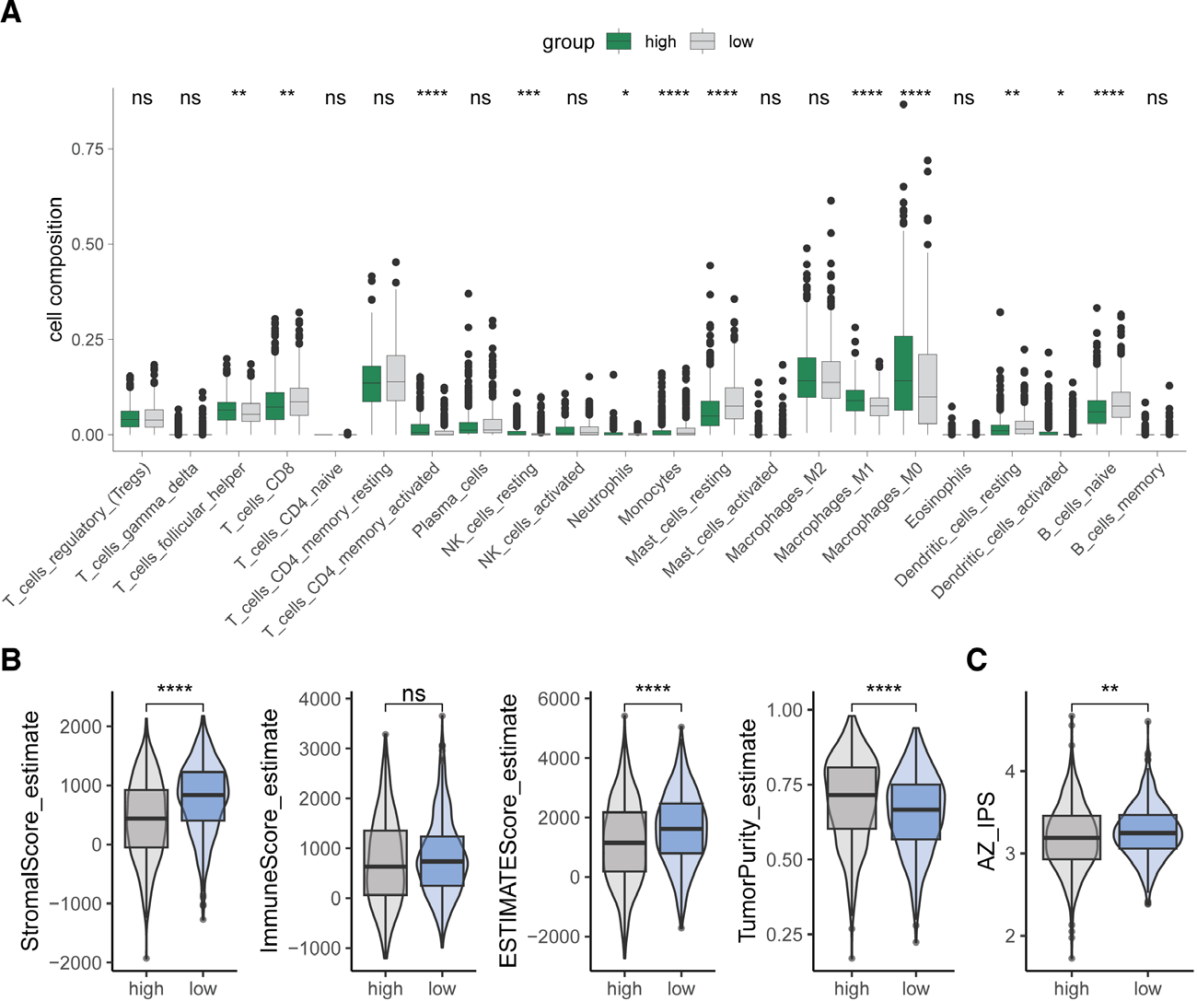
Association of the ARG-derived risk signature with tumor microenvironment (A) Comparison of the immune infiltration between the high- and low-risk groups. (B) Comparison of stromalscore, immunescore, ESTIMATEscore, and tumor purity between high- and low-risk groups. (C) Comparison of AZ_IPS between high- and low-risk groups. ns, not significant, *P < .05, **P < .01, ***P < .001, ****P < .0001.

**Figure 8.**
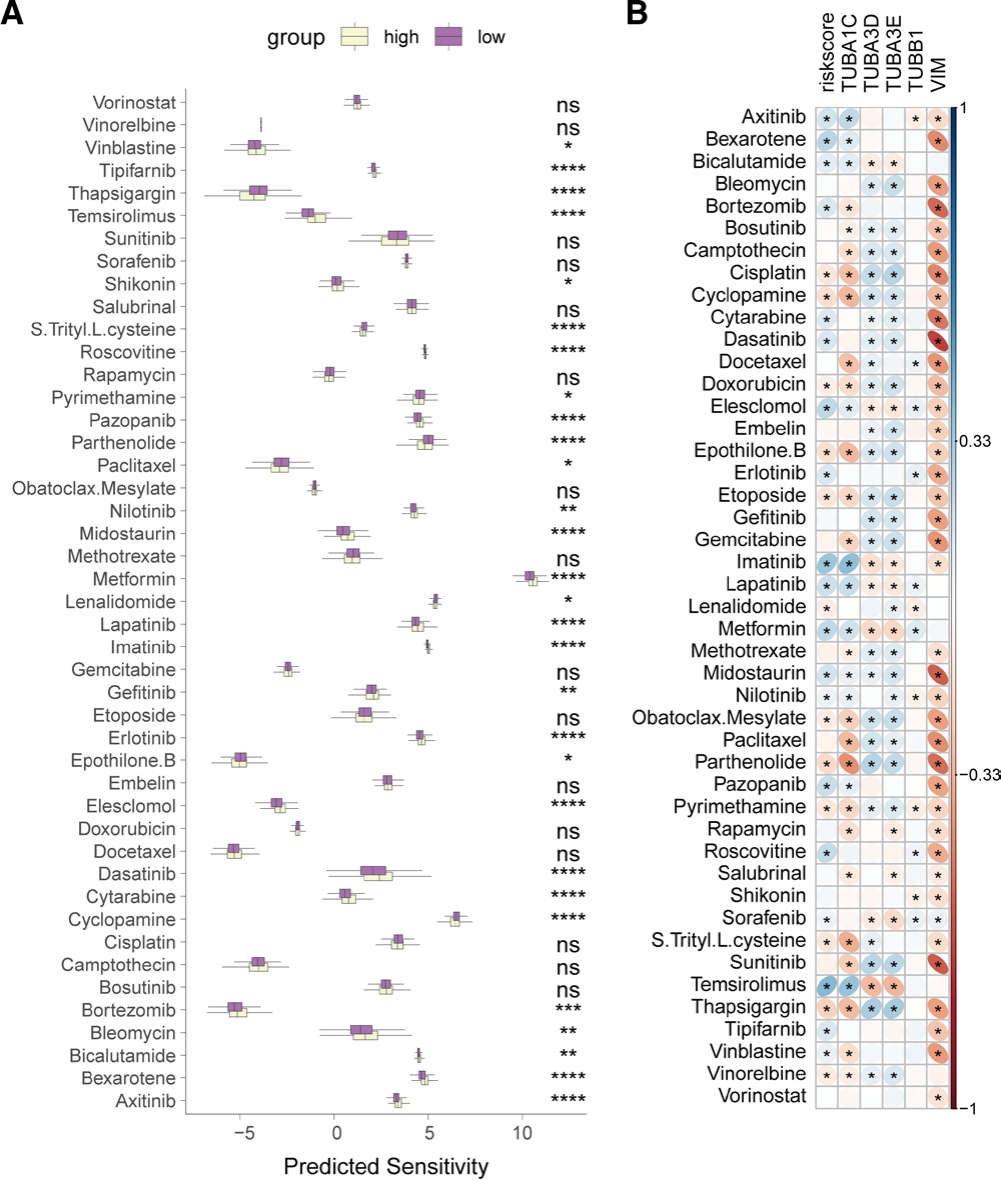
Association of the ARG-derived risk signature with drug sensitivity. (A) Comparison of drug sensitivity between high- and low-risk groups. (B) Heatmap showing the correlation between the risk signature and its constituent genes with drug sensitivity. ns, not significant, *P < .05, **P < .01, ***P < .001, ****P < .0001.

Immune infiltration analysis revealed that the ARG-based risk feature is related to tumor immune cell infiltration. Compared to the high-risk group, the low-risk group showed higher infiltration of CD8 + T cells, monocytes, resting mast cells, resting dendritic cells, and naive B cells, along with lower infiltration of follicular helper T cells, activated CD4 + memory T cells, neutrophils, M0 and M1 macrophages, and activated dendritic cells (Fig. 7A). Additionally, the risk score and its associated genes demonstrated significant correlations with the infiltration of certain immune cells (Figure S2). http://links.lww.com/MD/N721 Compared to the high-risk group, the low-risk group had higher stromalscores, ESTIMATEscores, and lower tumor purity (Fig. 7B). Concurrently, the low-risk group exhibited higher AZ_IPS scores than the high-risk group (Fig. 7C). Drug sensitivity analysis indicated that there were differential sensitivities to 29 drugs between high-risk and low-risk patients (Fig. 8A). Correlation analysis showed that *TUBA1C* and *VIM* were negatively correlated with the sensitivity to most drugs, whereas *TUBA3D* and *TUBA3E* were positively correlated with sensitivity to many drugs (Fig. 8B). This suggests that low-risk BC patients may potentially benefit more from immunotherapy, as higher AZ_IPS scores often correlate with better responses to anti-PD-1/PD-L1 treatments.

### 3.7. Nomogram incorporating aggrephagy-related genes and clinicopathological features

**Figure 9.**
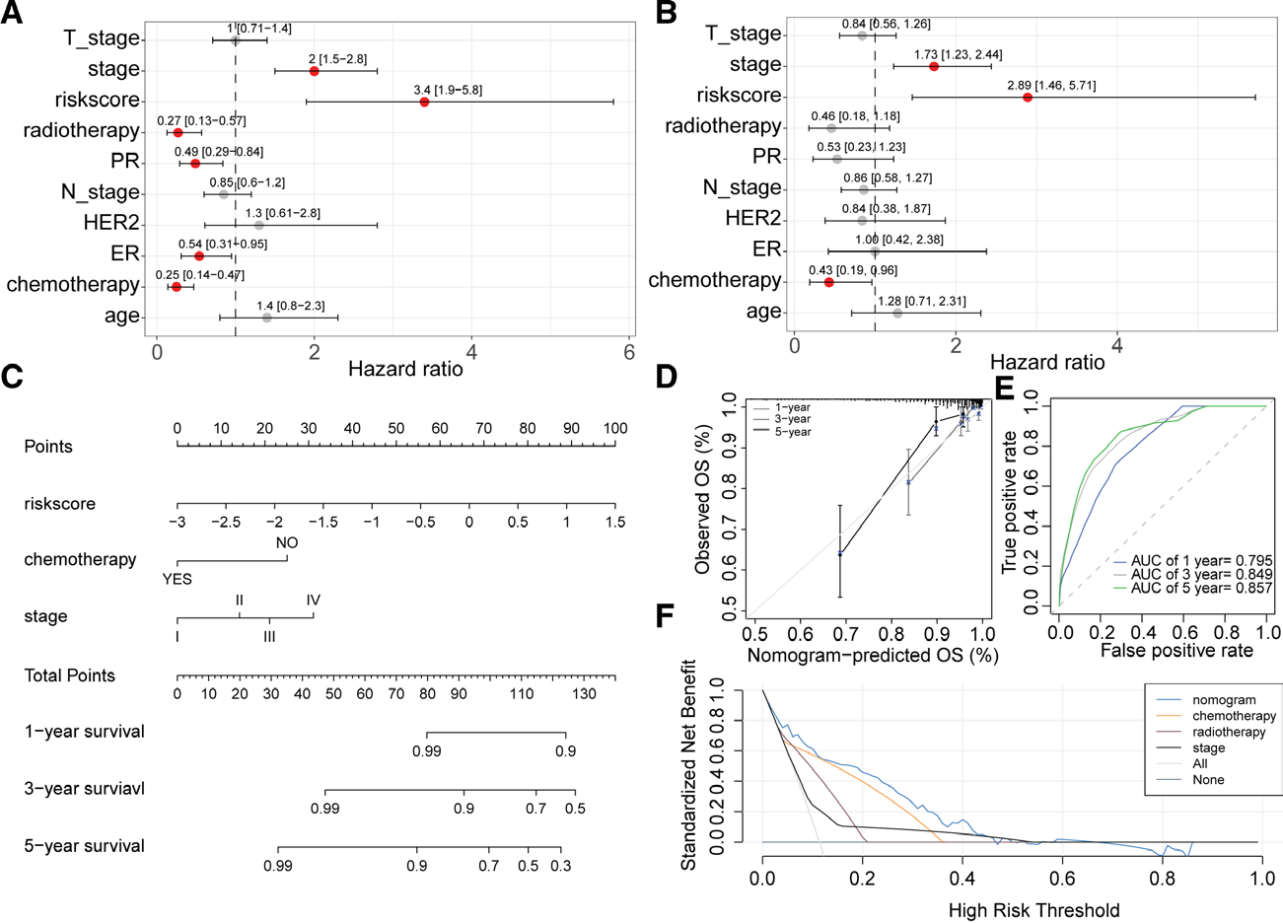
Development of a nomogram based on the ARG-derived risk signature and clinical-pathological features. (A) Univariate cox analysis identifying prognostic factors for BC patients. (B) Multivariate cox analysis identifying independent prognostic factors for BC patients. (C) Nomogram based on risk score, chemotherapy, radiotherapy, and stage. (D) Calibration curve of the nomogram predicting 1-, 3-, and 5-year overall survival. (E) Time-dependent ROC curves of the nomogram predicting 1-, 3-, and 5-year overall survival. (F) Decision curve analysis comparing the nomogram with other prognostic factors.

Univariate Cox analysis identified stage, risk score, radiotherapy, PR, chemotherapy, and ER as prognostic factors for BC (Fig. 9A). Further, multivariate cox analysis confirmed that stage, risk score, and chemotherapy were independent prognostic factors for BC (Fig. 9B). Consequently, a nomogram was constructed consisting of these independent prognostic factors to estimate 1-, 3-, and 5-year overall survival probabilities for patients (Fig. 9C). Calibration curves demonstrated that the nomogram provided good agreement between predicted and actual survival rates for 1-, 3-, and 5-year overall survival (Fig. 9D). Moreover, the nomogram achieved AUC values of 0.795, 0.849, and 0.857 for predicting 1-, 3-, and 5-year overall survival, respectively (Fig. 9E). The nomogram displayed a higher standardized net benefit compared to other prognostic factors when predicting prognosis (Fig. 9F).

### 3.8. Validation of aggrephagy-related gene-related gene expression

**Figure 10.**
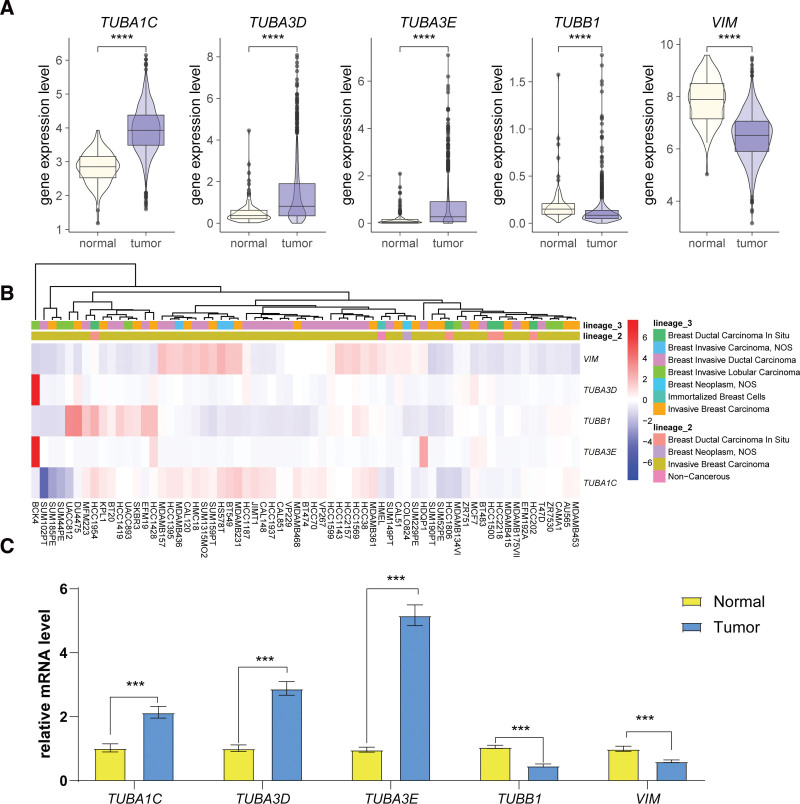
Validation of the expression patterns of ARG-derived risk signature-related genes. (A) Comparative expression analysis of ARG-derived risk signature-related genes between cancerous and normal tissues in the TCGA-BRCA cohort. ****P < .0001. (B) Heatmap representing the expression profiles of ARG-derived risk signature-related genes across BC cell lines and noncancerous breast cell lines based on the CCLE database. (C) Comparison of gene expression of 5 ARGs between BC tissue and adjacent normal tissues from 5 patients. ***P < .001, ****P < .0001.

To evaluate the expression patterns of the genes constituting the risk signature, we initially compared their expressions in 113 normal tissues against 1113 cancerous tissues from the TCGA-BRCA cohort. The results showed that *TUBA1C*, *TUBA3D*, and *TUBA3E* were significantly overexpressed, while *TUBB1* and *VIM* were significantly underexpressed in cancerous tissues (Fig. 10A). Additionally, the expression profiles across different BC cell lines derived from the CCLE database revealed heterogeneity in the expression of these genes (Fig. 10B). Specifically, *TUBA3D* and *TUBA3E* showed abnormally high expression in breast invasive lobular carcinoma cell lines, whereas the other 3 genes displayed either low or high expression in varying contexts. This suggests that these genes may have distinct clinical implications in different BC cell lines and possibly in diverse BC subtypes. In addition, clinical samples were used to validate the expression of the characteristic genes. It was found that *VIM* and *TUBB1* were significantly downregulated in BC tissues compared to adjacent normal tissues, while *TUBA1C*, *TUBA3D*, and *TUBA3E* were significantly upregulated in BC tissues (Fig. 10C).

## 4. Discussion

The traditional staging system for BC has become inadequate in meeting the evolving demands of personalized treatment approaches. Molecularly-informed prognostic models offer a diversified array of alternatives. In this study, we have, for the first time, constructed a prognostic risk signature and corresponding molecular subtypes for BC based on ARGs, which exhibit significant disparities in prognosis, clinicopathological features, and treatment responsiveness, underscoring their potential utility in individualized therapeutic strategies for BC patients. Ultimately, the nomogram incorporating the risk signature derived from ARGs and other clinicopathological characteristics demonstrated exceptional performance in predicting overall survival. The expression of the risk signature-related genes was also validated in additional clinical samples.

Protein aggregates have a close relationship with cancer development. In BC, certain mutations in the tumor suppressor gene *p53* have been shown to lead to protein misfolding and accumulation within cells.^[12]^ The normal *p53* protein plays a pivotal role in maintaining genomic stability, regulating the cell cycle, and inducing apoptosis.^[13]^ When *p53* loses its functional integrity due to mutation and forms aggregates, it not only forfeits its tumor-suppressive capabilities but might also trigger the aggregation of additional proteins, thereby promoting carcinogenesis. In gastric and colorectal cancers, *mTorc1* protein aggregates associated with inflammation have been found to activate intracellular signaling pathways, stimulating tumor growth.^[14,15]^ Aggrephagy is the autophagic process responsible for clearing protein aggregates within cells; disruption of this process can lead to the accumulation of detrimental protein aggregates, potentially exacerbating cancer progression or enhancing tumor invasiveness.^[16]^

Within the risk signature associated with ARGs, 5 key genes are implicated: *VIM*, *TUBB1*, *TUBA3E*, *TUBA3D*, and *TUBA1C. VIM* encodes an intermediate filament protein predominantly expressed in mesenchymal cells and contributes to the assembly and maintenance of the cellular cytoskeleton. In cancer research, Vimentin is often used as a marker for epithelial-mesenchymal transition (EMT).^[17]^ EMT represents a critical step by which tumor cells acquire migratory and invasive abilities, and it is associated with metastasis and poor prognosis in BC.^[18]^ Cell surface vimentin is recognized as an independent poor prognostic factor in colorectal cancer.^[19]^ *TUBB1* encodes β-tubulin 1, a member of the tubulin family that participates in spindle formation during cell division and other essential cellular processes.^[20]^ Mutations or expression abnormalities in tubulins may disrupt cell cycle regulation and mitosis, thereby promoting tumor initiation and progression.^[21]^ *TUBA3E* and *TUBA3D* encode different α-tubulin isoforms that play crucial roles in intracellular architecture, cell migration, and signal transduction. Analogous to *TUBB1*, variations or changes in expression of these tubulin isoforms may impact the behavior of tumor cells, such as proliferation and motility.^[22]^ *TUBA1C* is an α-tubulin gene with extensive research demonstrating its abnormal expression in cancer and its significant correlation with cancer prognosis.^[23]^ High expression of *TUBA1C* is identified as an independent risk factor for poor prognosis in BC.^[24]^ Studies have shown that knockdown of *TUBA1C* can inhibit tumor cell proliferation and migration, leading to apoptosis and G2/M phase arrest.^[25]^ Thus, functional abnormalities in these genes could influence the development and progression of BC through interference with cytoskeletal stability, cell cycle control, cell migration, and proliferation.

The tumor immune microenvironment is critical for determining responses to immunotherapy, with studies revealing that immune cells such as macrophages, T cells, and neutrophils release extracellular enzymes, angiogenic factors, and chemotactic cytokines within the tumor microenvironment, processes known to promote tumor growth.^[26,27]^ Moreover, the distribution and activity of immune cells within tumors decisively impact the effectiveness of cancer immunotherapies.^[28]^ Our findings demonstrate that the ARG-derived risk signature is significantly associated with immune cell infiltration in BC patients’ tumors. This suggests that different risk groups may correspond to distinct states of the tumor immune microenvironment, influencing tumor growth, progression, and response to therapy. Furthermore, the association between the ARGs-derived risk signature and drug sensitivity implies that personalized chemotherapy regimens are necessary for different risk groups to improve efficacy and reduce toxicity. Importantly, we observe that the expression levels of *TUBA1C* and *VIM* genes are negatively correlated with sensitivity to multiple drugs, indicating that tumors with high expression of these genes may be less sensitive or more resistant to these treatments. Conversely, the expression of *TUBA3D* and *TUBA3E* genes is positively correlated with sensitivity to most drugs, suggesting that higher expression of these genes may enhance the sensitivity of tumors to specific drugs.

Despite our study’s elucidation of the correlations between ARGs and prognosis as well as treatment response in BC, several limitations persist. Firstly, the retrospective cohort design employed in this study has not been rigorously validated through prospective clinical trials, which inherently attenuates the strength of the results in terms of their applicability and generalizability to clinical practice. Secondly, the present investigation lacks comprehensive experimental biological data; thus, it does not delve deeply into the specific molecular pathways and regulatory mechanisms engaged by ARGs during the onset and progression of BC. The precise manner in which ARGs influence the biological behavior of BC cells, and the causal relationships they bear with pathological and physiological changes in BC, remain unverified through methodologies such as cell culture experiments, animal models, or biochemical assays.

## 5. Conclusion

In summary, this study constructed a 5 ARGs-based prognostic signature for BC, which can indicate patient prognosis, response to immunotherapy, and drug sensitivity. Furthermore, a nomogram was developed to facilitate clinical decision-making. However, these findings necessitate further substantiation through cutting-edge prospective cohorts and experimental biology-level data in future studies.

## Author contributions

**Conceptualization:** Chenbo Ye.

**Data curation:** Chenbo Ye.

**Formal analysis:** Chenbo Ye.

**Funding acquisition:** Chenbo Ye.

**Investigation:** Chenbo Ye.

**Methodology:** Chenbo Ye.

**Project administration:** Chenbo Ye.

**Resources:** Chenbo Ye.

**Software:** Chenbo Ye.

**Supervision:** Chenbo Ye.

**Validation:** Chenbo Ye.

**Visualization:** Chenbo Ye.

**Writing – original draft:** Chenbo Ye.

**Writing – review & editing:** Chenbo Ye.

## Supplementary Material


