# Supplementary figures and images for: Exploring the role of aggrephagy-related signatures in immune microenvironment, prognosis, and therapeutic strategies of breast cancer
Source: Medicine (Baltimore). 2024 Oct 18;103(42):e39999. doi: 10.1097/MD.0000000000039999 (PMC11495756; doi:10.1097/MD.0000000000039999)

(A)

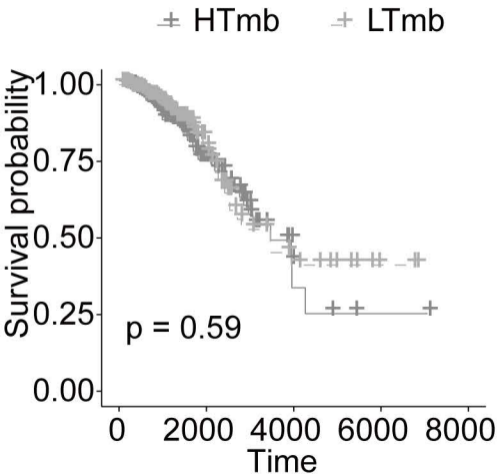

(B)

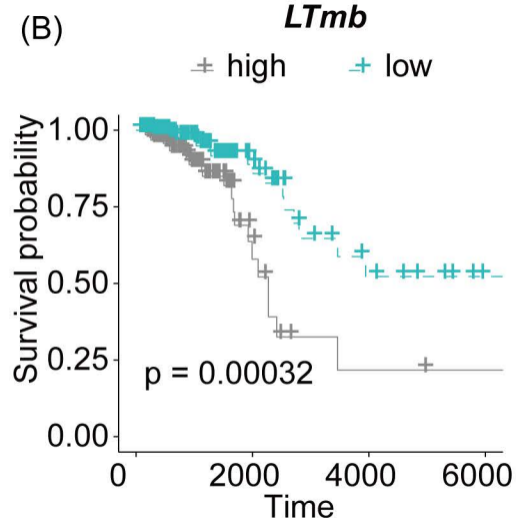

(C)

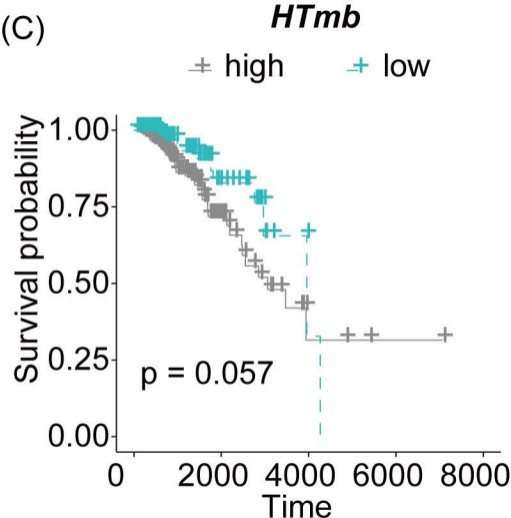

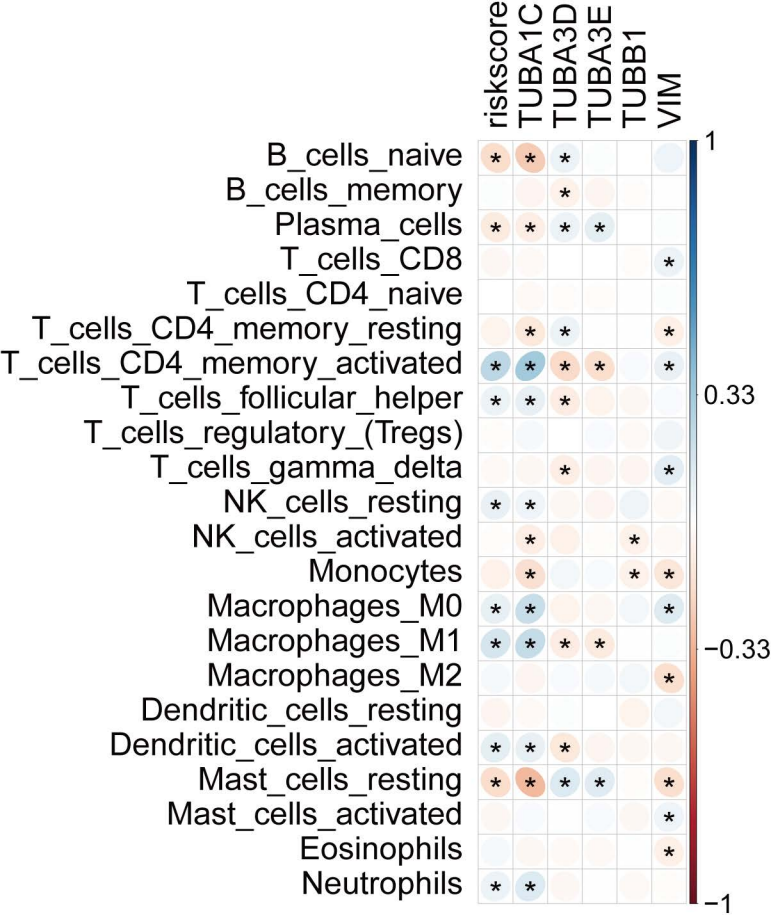

Supplement: Supplementary file 2 [file medi-103-e39999-s002.pdf]
